# Fermented Legumes as a Clean-Label Strategy for Shelf-Life Extension of Flatbread Under Modified Atmosphere Packaging: A Pilot-Scale Validation

**DOI:** 10.3390/foods15193368

**Published:** 2026-09-22

**Authors:** Simona Carbone, Luana Nionelli, Giuseppe Perri, Lorenzo Ciraldo, Federico Rametta, Andrea Minisci, Michela Verni, Carlo Giuseppe Rizzello, Erica Pontonio

**Affiliations:** 1 Department of the Soil, Plant and Food Science DiSSPA, University of Bari Aldo Moro, Via Amendola, 165/a, 70126 Bari, Italy; simona.carbone@uniba.it (S.C.); lorenzo.ciraldo@uniba.it (L.C.); federico.rametta@uniba.it (F.R.); erica.pontonio@uniba.it (E.P.); 2 Vallefiorita s.r.l., Viale Delle Comunicazioni 24, 72017 Ostuni, Italy; nionelli@vallefiorita.it (L.N.); a.minisci@vallefiorita.it (A.M.); 3 Department of Human Science and Quality of Life Promotion, San Raffaele University, Rome Italy, Via di Val Cannuta, 247, 00166 Rome, Italy; michela.verni@uniroma1.it; 4 Department of Environmental Biology, “Sapienza” University of Rome, 00185 Rome, Italy; carlogiuseppe.rizzello@uniroma1.it

**Keywords:** legumes, lactic acid bacteria, antifungal activity, flatbread, modified atmosphere packaging, shelf Life

## Abstract

This study investigated fermentation-mediated antifungal activity across different legume matrices and their potential for extending the shelf life of legume-based bakery products. Chickpea, lentil, pea, and grass pea flours were fermented using selected lactic acid bacteria and yeasts. Water/salt-soluble fractions from fermented doughs were screened against bakery spoilage fungi, revealing marked substrate- and starter-dependent antifungal activity. The most promising formulations were incorporated into focaccia-type flatbreads and evaluated for 60 days under CO_2_/N_2_ modified atmosphere packaging at pilot scale. Compared with their non-fermented counterparts, grass pea fermented with *Levilactobacillus brevis* AM7 maintained mold populations below the detection limit (<2 Log CFU g^−1^) throughout storage, with visible mold detected in 5% of packages at day 60. Pea fermented with *Lactiplantibacillus plantarum* 1A7 maintained mold populations below the detection limit up to day 45, with approximately 3% of packages showing visible mold at day 60. These two formulations showed the strongest antifungal performance among the fermented legume formulations, approaching the protection provided by the calcium propionate-containing wheat control, which showed the highest level of fungal protection. Fermentation partially mitigated the increase in hardness throughout storage, particularly in grass pea-based flatbreads. Overall, the findings highlight the importance of substrate–starter combinations in determining the antifungal performance of fermented legume ingredients and demonstrate their potential, when combined with modified atmosphere packaging, to improve the microbiological stability and shelf-life performance of selected legume-based flatbreads.

## 1. Introduction

White bread is one of the most widely consumed staple foods worldwide, and its wastage represents a significant challenge due to its limited shelf life, which is mainly constrained by staling and microbial spoilage [1]. Among microbial spoilage agents, fungal contamination—particularly species belonging to the genera *Penicillium* and *Aspergillus*, is a major cause of quality deterioration in bakery products, leading to economic losses, safety concerns, and reduced consumer acceptance [2].

To mitigate spoilage and extend shelf life, synthetic preservatives such as calcium propionate and ethanol have traditionally been used. However, increasing consumer demand for clean-label products, together with concerns regarding synthetic additives, has stimulated interest in alternative preservation strategies [3,4]. In this context, modified atmosphere packaging (MAP) has emerged as an effective technology for extending the shelf life of bakery products by replacing atmospheric air with tailored gas mixtures, typically enriched in carbon dioxide (CO_2_) and nitrogen (N_2_), which inhibit fungal growth and slow quality deterioration [5,6].

Despite its effectiveness, MAP does not always provide complete control of microbial spoilage, and its performance may be influenced by storage conditions and by the stability of the modified atmosphere over time [7]. As a result, recent research has focused on integrated preservation strategies combining MAP with complementary technologies such as active packaging, antimicrobial agents, UV irradiation, ozone treatment, and bioactive compounds, aiming to enhance both shelf life and food safety [7]. More broadly, current trends in food preservation are shifting towards multifunctional approaches designed not only to extend shelf life but also to maintain food quality, safety, and nutritional value [8].

Within this framework, plant-derived bioactive compounds have gained increasing attention as natural alternatives to synthetic preservatives. Essential oils, phenolic compounds, alkaloids, terpenes, and bioactive peptides have been widely investigated for their antimicrobial and antioxidant properties [9]. Among plant sources, legumes, which are widely recognized for their contribution to the sustainability of agricultural systems through biological nitrogen fixation and reduced dependence on synthetic nitrogen fertilizers [10], have emerged as a valuable source of proteins, bioactive peptides, polyphenols, and other compounds associated with antifungal activity [11,12]. However, many antifungal compounds present in legumes are protein-bound or latent within native structures, which limits their direct bioactivity [11]. Bioprocessing strategies, including enzymatic hydrolysis and fermentation, have been investigated to enhance the release or generation of legume-derived bioactive compounds with antifungal properties [13]. In particular, fermentation with selected lactic acid bacteria and yeasts has been shown to promote the production of bioactive metabolites capable of inhibiting bakery spoilage fungi, with effects comparable under certain conditions to conventional preservatives [14]. Despite the growing interest in both fermented legumes and MAP, to our knowledge, evidence on the combined use of selected fermented legume ingredients and modified-atmosphere packaging for extending the shelf life of focaccia-type flatbread remains limited, particularly under pilot-scale production conditions. Therefore, the present study aimed to investigate whether substrate-tailored legume fermentation, integrated with MAP, could improve the microbiological stability and overall quality of focaccia-type flatbread during extended storage. To this end, a two-step experimental approach was adopted. First, four legume flours (lentil, grass pea, chickpea, and pea) were fermented using selected lactic acid bacteria and yeasts to produce legume sourdoughs, which were screened in vitro against a broad panel of bakery spoilage fungi. Based on their antifungal performance, the most promising starter–legume combinations were selected and transferred to the second phase of the study, where fermentation processes were scaled up under industrial-like conditions. The resulting sourdoughs were incorporated into focaccia formulation, a traditional Italian flatbread widely produced and commercialized at industrial scale [15]. The final products were packaged under modified atmosphere conditions and monitored for 60 days to evaluate their chemical, biochemical, microbiological, and textural stability. Unfermented legume formulations and a calcium propionate-containing wheat formulation were included as comparative controls to assess the specific contribution of legume fermentation to preservation.

## 2. Materials and Methods

### 2.1. Legume Grains and Flour Production

Whole grains of lentil (*Lens culinaris* L.), grass pea (*Lathyrus sativus* L.), chickpea (*Cicer arietinum* L.), and pea (*Pisum sativum* L.) were supplied by Terre di Altamura s.r.l. (Altamura, Bari, Italy). Legume grains were milled using an electric stone mill (MOD. MB250) connected to a centrifugal pulsation tumbler (Partisani Combi Junior, Partisani S.r.l., Forlì, Italy), obtaining flours with a particle size below 500 μm. The resulting flours were subsequently used for sourdough production. The proximate composition of the selected legumes, as provided by the manufacturer, is reported in Table S1.

### 2.2. Microorganisms and Culture Conditions

The lactic acid bacteria (LAB) *Lactiplantibacillus plantarum* 1A7 and *Levilactobacillus brevis* AM7 and the yeast *Wickerhamomyces anomalus* LCF1695, belonging to the DiSSPA Culture Collection, were used as starter cultures for legume-based sourdough production. LAB strains were grown in De Man, Rogosa, and Sharpe (MRS) broth (Oxoid) for 16 h at 30 °C, whereas *W. anomalus* was grown in Malt Extract Broth (Oxoid) for 48 h at 25 °C. Before inoculation, cells were harvested by centrifugation (10,000× *g*, 10 min, 4 °C), washed twice with 50 mM phosphate buffer (pH 7.0), and resuspended in tap water. The fungal strains used for the hyphal radial growth inhibition assay are reported in Table 1. The fungal collection included strains belonging to the DiSSPA Culture Collection, isolates recovered from raw material storage areas and processing and storage facilities at the Vallefiorita S.r.l. (Ostuni, Italy) plant, and reference strains obtained from the Leibniz Institute DSMZ–German Collection of Microorganisms and Cell Cultures GmbH (Braunschweig, Germany). Before use, fungal strains were subcultured on Potato Dextrose Agar (PDA) plates and incubated at 25 °C for 7 days.

### 2.3. Production of Legume-Based Sourdough (LS)

Each legume flour was mixed with tap water to obtain a dough yield (DY; dough weight × 100/flour weight) of 330, corresponding to a flour-to-water ratio of approximately 30:70 (*w*/*w*). Doughs were mixed for 5 min using a high-speed mixer (IM 58 Mecnosud, Flumeri, Italy). For each legume flour, four fermentation condition were performed. Three doughs were individually inoculated with either *Lp. plantarum* 1A7, *Lev. brevis* AM7, or *W. anomalus* LCF1695, whereas a fourth dough was inoculated with the three strains simultaneously (COMB). Cell suspensions were adjusted to obtain initial populations of 7.0 Log CFU g^−1^ for LAB and 6.0 Log CFU g^−1^ for yeast. Cell density was estimated by measuring optical density at 620 nm (OD620) for LAB and at 600 nm (OD600) for yeast. An OD620 value of 2.5 corresponded to 9.0 Log CFU mL^−1^ for LAB, whereas an OD600 value of 0.1 corresponded to 6.0 Log CFU mL^−1^ for yeast. All sourdoughs were incubated at 30 °C for 24 h. After fermentation, samples were collected for microbiological, physicochemical, and biochemical analyses.

### 2.4. Microbiological, Chemical and Biochemical Monitoring of Sourdough Fermentation

Microbial counts, pH, and total titratable acidity (TTA) were determined before and after fermentation. Presumptive LAB were enumerated on MRS agar (Oxoid) supplemented with cycloheximide (0.1 g/L) and incubated at 30 °C for 48 h. *W anomalus* was enumerated on Malt Extract Agar (MEA, Oxoid) incubated at 25 °C for 48 h. The pH was measured using a pH meter (Model 507, Crison, Milan, Italy) equipped with a food penetration probe. TTA was determined by homogenizing 10 g of dough with 90 mL of distilled water and expressed as the volume (mL) of 0.1 M NaOH required to reach pH 8.3, according to Perri et al., 2024 [16].

### 2.5. Water/Salt-Soluble Extracts of Legume-Based Sourdough

Water/salt-soluble extracts (*w*LS) from each sourdough were prepared according to the method originally described by Weiss et al. (1993) [17], with some modifications. Legume-based sourdough samples were suspended in 50 mM Tris-HCl buffer (pH 8.8) at a ratio of 1:4 (sample dry weight to solution volume, *w*/*v*), maintained at 4 °C for 1 h, and stirred every 15 min. The suspensions were centrifuged at 11,000× *g* for 10 min, and the resulting supernatants were collected as *w*LS. Extracts were also prepared from the corresponding unfermented legume flours following the same procedure. These extracts were used as fermentation controls to assess the contribution of fermentation to biochemical modifications and were included in the hyphal radial growth inhibition assay.

### 2.6. Biochemical Characterization of wLS

*w*LS obtained before and after fermentation were analysed for peptide content, total free amino acids (TFAA), and organic acids. Proteolysis was evaluated by quantifying TFAA and peptides using the ninhydrin- and o-phthaldialdehyde-based assays described by Doi et al. (1981) [18] and Church et al. (1985) [19], respectively. Lactic and acetic acid concentrations were quantified using the K-DLATE and K-ACET enzymatic kits (Megazyme International Ireland Limited, Bray, Ireland), respectively, according to the manufacturer’s instructions.

### 2.7. Hyphal Radial Growth Rate

For the hyphal radial growth inhibition assay, *w*LS were sterilized by sequential vacuum filtration through membrane filters with progressively decreasing pore sizes, down to a final pore size of 0.2 μm (Millipore Corporation, Bedford, MA, USA), before use. The antifungal activity of each *w*LS was evaluated by measuring fungal radial growth according to the method described by Quiroga et al. (2001) [20], with some modifications. Sterile *w*LS were incorporated into PDA at a final concentration of 33% (*v*/*v*). Aliquots (9 mL) of the supplemented medium were poured into sterile Petri dishes (60 mm diameter), and 5 μL of standardized conidial suspensions (adjusted to 10^4^ conidia/mL in sterile distilled water) were inoculated at the centre of each plate. Plates were incubated aerobically at 25 °C for 7 days. Three independent replicates were performed for each condition. Radial mycelial growth was determined by measuring colony diameter after incubation, calculated as the mean of four measurements taken along different axes for each colony. Control plates were prepared by replacing the *w*LS fraction with 50 mM Tris-HCl buffer (pH 8.8) at the same ratio used for *w*LS incorporation (67:33, *v*/*v*) and represented the reference growth conditions. Both fermented and unfermented legume *w*LS were evaluated against this control to determine their antifungal activity and to assess the specific contribution of fermentation to fungal growth inhibition. The percentage of growth inhibition was calculated according to the following equation: Inhibition (%) = [(C − T)/C] × 100, where C represents fungal growth (colony diameter, mm) under control conditions (PDA supplemented with Tris-HCl buffer) and T represents fungal growth in the presence of *w*LS.

### 2.8. Pilot-Scale Production of Selected Legume-Based Sourdoughs

Based on the results obtained from the in vitro antifungal screening, the most promising starter–legume combinations were selected for scale-up evaluation. Specifically, *Lp. plantarum* 1A7 sourdoughs produced from chickpea, pea, and lentil flours, and *Lev. brevis* AM7 sourdough produced from grass pea flour were selected for further evaluation. The selected legume-based sourdoughs were produced at pilot scale using industrial fermentation systems (Novasilos SNL100, Forlì, Italy) under the same fermentation conditions adopted at laboratory scale.

### 2.9. Pilot-Scale Production of Focaccia-Type Flatbreads and Modified Atmosphere Packaging

Table 2 reports the formulations of focaccia-type flatbread prepared according to the industrial protocol. The production trials were carried out at the industrial facilities of Vallefiorita S.r.l. (Italy) following the company’s standard manufacturing procedures. Doughs contained wheat flour (type 0), durum wheat semolina, water, baker’s yeast, salt, extra virgin olive oil, and barley malt. Four formulations were produced: wheat-based focaccia (CWF, control wheat focaccia), calcium propionate-containing wheat focaccia (CWF-P), fermented legume focaccia (FLF), and control legume focaccia (CLF). CWF was prepared using only wheat flour (type 0) and durum wheat semolina as the flour fraction. CWF-P was prepared according to the same formulation as CWF, with the addition of calcium propionate (0.3%, *w*/*w*, based on flour weight) as a conventional antifungal preservative. FLF formulations were obtained by incorporating selected legume-based sourdoughs (DY = 330) at 33% (*w*/*w*) of the final dough weight. As reported in Table 2, this sourdough addition contributed 10% legume flour and 23% water on a dough basis. The 10% legume flour corresponded to 15.3% of the total flour content on a flour basis and replaced an equivalent proportion of the wheat flour and durum wheat semolina fraction used in the control formulation, while maintaining the total flour content at 65% (dough basis). The sourdoughs were prepared from pea, chickpea, and lentil flours fermented with *Lp. plantarum* 1A7, and grass pea flour fermented with *Lev. brevis* AM7. CLF formulations were prepared using the corresponding unfermented legume flour (10% of dough weight) and additional water (35% of dough weight), thereby matching the total legume flour and water contents of the corresponding FLF formulations. All doughs were standardized to a final dough yield (DY; dough weight × 100/flour weight) of 153.

The ingredients were mixed using a spiral mixer (Pietroberto, Piovene Rocchette, Italy) at low speed (80 rpm) for 5 min and subsequently at high speed (160 rpm) for 10 min. Doughs were divided into pieces of 100 g using a volumetric dough divider (Artezen, Schio, Italy), rounded using a conical dough rounder (Gerosa, Valmadrera, Italy), and placed into PTFE-coated molds (diameter 10.5 cm). Dough pieces were proofed at 37 ± 1 °C and 85% relative humidity in a proofing chamber (Ing. Polin & C., Verona, Italy) for 30 min. Subsequently, the dough surface was manually dimpled, as typically performed for focaccia, and the samples were returned to the proofing chamber under the same conditions for a further 1 h and 15 min. All samples were baked in an electric rotor oven (Ing. Polin & C., Verona, Italy) at 215 °C for 10 min. After baking, flatbread was cooled at room temperature and subsequently packaged. For each focaccia formulation, two independent production trials were carried out. In each trial, 40 focaccia-type flatbreads were produced and individually packaged under modified atmosphere conditions. Specifically, 40 CWF, 40 CLF (for e ach legume flour), and 40 FLF (for each corresponding selected legume sourdough: chickpea, pea, lentil and grass pea) were produced per trial. Therefore, 80 individually packaged flatbreads were obtained for each focaccia formulation across the two independent trials. Packaging was carried out under modified atmosphere (50% N_2_ and 50% CO_2_) using a thermoforming machine (Veripack, Gorla Maggiore, Italy). The packaging material consisted of high-barrier multilayer thermoforming films. The bottom film was a coextruded multilayer structure based on polyamide (PA), ethylene vinyl alcohol (EVOH), and polyethylene (PE), while the top film consisted of biaxially oriented polyester (PET) combined with a coextruded barrier layer of polyethylene (PE), EVOH, and metallocene polyethylene (m-PE). After packaging, the flatbreads were stored at ambient temperature in the company’s storage facilities for 60 days.

### 2.10. Shelf-Life Monitoring of Focaccia-Type Flatbread

#### 2.10.1. Microbiological, Chemical and Biochemical Aspects

Shelf-life was evaluated during storage for 60 days. Analyses were performed at 0, 15, 30, 45, and 60 days of storage. For microbiological analyses, 10 g of focaccia sample were suspended in 90 mL of sterile sodium chloride solution (0.9%, wt/vol) and homogenized in a Stomacher lab blender for 2 min at room temperature. For molds enumeration, 100 µL of each dilution were inoculated by the spread-plate method onto Potato Dextrose Agar (PDA, Oxoid) supplemented with chloramphenicol (0.1 g/L) and incubated at 25 °C for 96 h. Based on the plating of 100 µL of the 10^−1^ suspension, the detection limit of the method was 100 CFU g^−1^ (2 Log CFU g^−1^). Samples showing no fungal colonies at the 10^−1^ dilution were reported as <100 CFU g^−1^ (<2 Log CFU g^−1^). Moisture content was determined using a thermogravimetric moisture analyzer (Ohaus MB25 Moisture Analyzer, Ohaus Corporation, Parsippany, NJ, USA). 5 g of sample were placed on an aluminium sample pan and dried at 105 °C until constant weight was reached. Moisture percentage was automatically calculated by the instrument software and expressed as % moisture (*w*/*w*). Water activity (aw) was determined at 25 °C using an AquaLab 4TE Dew Point water activity meter (Decagon Devices Inc., Pullman, WA, USA), according to Pontonio et al. (2019) [21]. pH and TTA were determined according to the methods described in Section 2.4.

#### 2.10.2. Visible Mold Growth Assessment

Visible mold growth was monitored every 5 days on the individually packaged focaccia-type flatbreads throughout the 60-day storage period. The experiment comprised two independent production trials, with 40 packages prepared per trial. Of the 40 packages from each trial, 15 were destructively sampled for physicochemical analyses over time (3 packages × 5 sampling times), whereas the remaining 25 unopened packages were continuously monitored for visible mold growth. Thus, 50 packages per formulation were monitored for mold growth across the two independent production trials (25 packages per trial). At each sampling time, these packages were visually inspected for the presence of fungal growth on the product surface. The presence of any visible mold growth within a package, regardless of the extent of colonization, was considered sufficient to classify the package as mold-positive. For each production trial, the percentage of mold-positive packages was calculated at each monitoring time as the number of packages that had developed visible mold by that time divided by the 25 packages monitored in that trial. Once a package was classified as mold-positive, it remained classified as positive at all subsequent sampling times. Mold occurrence was therefore expressed as the cumulative percentage of mold-positive packages over storage time.

#### 2.10.3. Texture Profile Analysis (TPA)

Texture profile analysis (TPA) was carried out at 0, 15, 30, 45, and 60 days of storage using a Imada FRTS-100N Texture Analyzer (Imada, Toyohashi, Japan) equipped with a spherical probe (20 mm diameter) following the method described by Montemurro et al. (2024) [22]. Measurements were performed on whole focaccia samples (diameter 10.5 cm). Samples were subjected to a double compression cycle with a test speed of 1 mm s^−1^ and a deformation of 40% of the original sample height. The force–time curves obtained were used to calculate hardness parameter.·

#### 2.10.4. Statistical Analysis

All fermentations were performed in three independent biological replicates (n = 3), which were considered the experimental units. Analytical measurements were conducted in triplicate for each biological replicate, and the technical replicates were averaged before statistical analysis. For the shelf-life study, two independent production batches were prepared for each formulation. At each storage time, three independently packaged focaccia-type flatbreads were analysed within each production trial. The three packages from each production batch were averaged to obtain one value per production trial, and the two independent production batches were considered the experimental units (n = 2) for statistical analysis. Statistical analyses were performed using Statistica 12.5 (StatSoft Inc., Tulsa, OK, USA). Data are expressed as mean ± standard deviation (SD). For all analyses performed during the shelf-life study (i.e., moisture content, water activity, pH, TTA, TPA, and fungal counts), each formulation–storage time combination was considered as an individual treatment group. Differences among treatment groups were evaluated by one-way analysis of variance (ANOVA), followed by Tukey’s honestly significant difference (HSD) test when significant differences were detected. Statistical significance was set at *p* < 0.05. Visible mold occurrence was considered a descriptive shelf-life endpoint and was therefore not subjected to ANOVA. For the hyphal radial growth inhibition assay, three independent replicates were performed for each *w*LS–fungal strain combination (n = 3). Growth inhibition percentages were calculated for each replicate and individual inhibition values were visualized as a heatmap. For each legume–microorganism combination, the mean inhibition percentage across the entire fungal strain collection was then calculated from the three independent replicates and analysed by one-way ANOVA followed by Tukey’s HSD test (*p* < 0.05).

## 3. Results and Discussion

### 3.1. Set-Up and Monitoring of the Fermentation Process

Fermentation is increasingly recognized as a sustainable and versatile bioprocessing strategy for the valorization of non-conventional agri-food substrates and the generation of value-added compounds [23]. The starter cultures investigated in this study were selected based on previous evidence of their effectiveness in improving the microbiological stability and shelf life of cereal- and legume-based fermented bakery products. The combination of *Lp. plantarum* 1A7 and *W. anomalus* LCF1695 has been shown to enhance antifungal activity in wheat bread through the synergistic production of organic acids, antifungal peptides, and volatile compounds [24]. Similarly, the use of *W. anomalus* in combination with lactic acid bacteria contributed to prolonged microbiological stability in sourdough-based panettone [25], while *Lev. brevis* AM7 demonstrated broad-spectrum antifungal activity in faba bean fermentation, delaying mold development in composite bread [26]. On this basis, the present study evaluated these microbial strains individually and in combination in legume-based dough systems to assess their antifungal potential and to elucidate the contribution of each microbial component to the overall bioactivity.

As shown in Table 3, all starter microorganisms showed good adaptation to the different legume substrates, as demonstrated by increases of 2.20–2.60 Log CFU g^−1^ after 24 h of fermentation. Final populations reached 9.20–9.91 Log CFU g^−1^ for LAB and 6.25–8.76 Log CFU g^−1^ for *W. anomalus*, indicating that all tested legume matrices supported microbial growth. Fermentation induced significant acidification (*p* < 0.05), with pH decreasing by 1.73–3.09 units and total titratable acidity increasing by 10.00–15.00 mL NaOH across the different legume substrates and starter cultures. This acidification was accompanied by a marked accumulation of organic acids. Lactic acid increased from initial levels of 0.57–2.70 mmol/kg to 89.99–112.96 mmol/kg after 24 h, whereas acetic acid also increased, although to a lower extent, reaching values between 2.89 and 19.81 mmol/kg (Table 3).

Overall, these results indicate that all legume matrices supported active fermentation, confirming their suitability as substrates for starter-driven fermentations in agreement with the previous literature [14,27,28,29]. The acidification observed in doughs inoculated with *W. anomalus* alone may be associated with the growth of indigenous lactic acid bacteria naturally present in the flour, whose populations increased by approximately 2 log cycles during fermentation [30].

Beyond acidification, fermentation also affected nitrogen metabolism and protein-derived compounds. Under conditions of limited carbohydrate availability, LAB metabolism is known to shift towards the utilization of peptides and amino acids as alternative energy and nitrogen sources through transamination and deamination pathways [31,32]. This metabolic flexibility is supported by their ability to hydrolyze proteins through extracellular proteinases, promoting the release of peptides and amino acids and contributing to a dynamic interaction between proteolysis and microbial utilization in protein-rich substrates [33]. Here, fermentation affected the proteolytic profile of all legume-based doughs. In yeast-only, LAB-inoculated, and combined systems, peptide concentrations generally decreased with the largest reduction observed in chickpea dough fermented with AM7 (−55%), whereas only minor changes were detected in grass pea fermented with 1A7 (−2%) (Table 3). In contrast, total free amino acids (TFAA) showed a more variable pattern, depending on both the legume substrate and the starter culture. Increases were observed in several fermentations, with AM7 promoting the highest accumulation, reaching up to +235% in grass pea doughs, in agreement with previous reports on its ability to enhance amino acid release in legume substrates [26,34].

### 3.2. In Vitro Antifungal Activity and Sourdough Selection

The antifungal activity of legume-based sourdoughs was first evaluated through an in vitro screening approach to assess the influence of both the fermenting microorganism and the legume substrate on mold growth inhibition. This approach was based on the ability of *w*LS from fermented matrices to inhibit the development of spoilage fungi most associated with baked goods spoilage (Table 1) [35,36]. Heatmap analysis (Figure 1a) revealed marked variability in fungal susceptibility to fermented legume extracts, with three main susceptibility clusters being identified. The first cluster included highly susceptible species, such as *Penicillium paneum*, *P. carneum*, *P. roqueforti*, and *Paecilomyces variotii*, which showed inhibition values generally ranging from 60 to 100%. A second cluster comprised intermediate-responsive *Penicillium* species, characterized by isolate-dependent inhibition patterns, whereas *Aspergillus flavus*, *A. niger*, and *A. clavatus* formed the least susceptible group, consistently showing limited inhibition by fermented legume extracts, in agreement with their well-documented tolerance to environmental stresses and antifungal compounds [37].

Overall, fermentation significantly reshaped the inhibitory spectrum of legume matrices compared with the respective unfermented CTR formulations (*p* < 0.05), as confirmed by the average inhibition calculated across the entire fungal collection. The extent of this effect depended on the specific substrate–starter combination (Figure 1b). The highest improvements compared with the corresponding unfermented controls were observed for *Lp. plantarum* 1A7 in pea (+34.2%) and chickpea (+30.2%) fermentations, and for *Lev. brevis* AM7 in grass pea (+33.4%). In contrast, lentil showed only limited changes after fermentation, with variations in the average inhibition ranging from −7.4% to +5.4% depending on the starter culture, despite displaying microbial growth and acidification patterns comparable to those observed in the other legume matrices (Table 3). Fermentations involving *W. anomalus* strain showed a substrate-dependent antifungal response, with only moderate increases in inhibition observed in pea (+16.1% for LCF) and grass pea (+8.3% and +10.8% for LCF and COMB, respectively), whereas no enhancement was detected in chickpea. This variability suggests that the expression of the *W. anomalus*-associated antifungal phenotype is strongly influenced by the fermentation matrix. Differences in substrate composition, including carbohydrate availability, may modulate yeast metabolism and the production of antifungal volatile compounds, such as ethyl acetate, previously associated with the activity of this strain [24,38].

Although the synergistic action of lactic and acetic acids produced during fermentation is generally considered a major contributor to the antifungal activity of LAB-fermented foods [39,40], the comparable levels of organic acids, pH, and TTA observed among several fermented legume matrices (Table 3) indicate that acidification alone cannot fully account for the different inhibition profiles. Legumes represent a natural reservoir of antifungal proteins and bioactive peptides, including defensins, lectins, chitinases, and storage protein-derived sequences, which may contribute to fungal growth inhibition [11]. During fermentation, microbial proteolytic activity may promote the release of encrypted bioactive peptides from storage proteins such as vicilins and legumins. Consistently, previous studies reported the generation of antifungal peptides in legume sourdough systems fermented with the same starter strains used in the present study, including faba bean fermented with *Lev. brevis* AM7 [26] and pea fermented with *Lp. plantarum* 1A7 [41]. Since no universal threshold of inhibition is available for radial growth assays and agar-based methods often provide a conservative estimation of antifungal activity [42], the most promising fermented sourdoughs for subsequent industrial-scale production of focaccia-type flatbreads were selected based on the average inhibition calculated across the fungal panel and the breadth of the inhibitory spectrum (Figure 1a,b). Chickpea fermented with *Lp. plantarum* 1A7 showed the highest average inhibition (55%), followed by pea fermented with *Lp. plantarum* 1A7 (50.18%) and grass pea fermented with *Lev. brevis* AM7 (50.10%). These formulations combined strong overall activity with a broad inhibitory spectrum, including complete inhibition of *Paecilomyces variotii* and substantial inhibition of several recalcitrant *Penicillium* isolates and *Aspergillus niger* strains. Although lentil fermented with *Lp. plantarum* 1A7 showed lower antifungal activity, it was selected for baking trials due to its comparable fermentation performance (Table 3), providing a useful model to in situ evaluate the specific contribution of sourdough-derived antifungal activity beyond the acidification.

### 3.3. Integrated Shelf-Life Monitoring of Focaccia-Type Flatbread Under Modified Atmosphere Packaging

#### 3.3.1. Physicochemical Properties

Following the in vitro screening, legume-based sourdoughs were incorporated to replace 15% (*w*/*w*) of semolina and soft wheat flour (Table 2), a substitution level previously identified as an optimal compromise between technological performance and nutritional improvement [43]. Moisture content, water activity (*aw*), and acidification parameters represent relevant variables for monitoring bread stability during storage under modified atmosphere packaging (MAP), as they are closely related to changes in physicochemical properties and microbial stability [6]. The use of CO_2_/N_2_ atmospheres has been shown to limit quality deterioration and microbial spoilage by preserving the physicochemical characteristics of bakery products [6,44,45]. Consistent with these observations, moisture content and *aw* remained largely unchanged throughout storage (Figure 2), indicating that the applied MAP conditions were effective in maintaining water-related properties during shelf life. Initial moisture content averaged 37%, with a 5% average decrease observed at the end of the monitoring period, with the most pronounced losses recorded in CWF-P (Figure 2). Similarly, *aw* remained largely stable during storage. At day 0, significant differences (*p* < 0.05) were observed among formulations, with legume-based flatbreads, particularly FLF_1A7-Pea, showing lower aw values (≈0.96) than the other samples. This behaviour could be related to changes in the hydration properties of fermented legume components, which may affect water distribution within the bread matrix, as previously reported for fermented pea-based ingredients [46,47]. Nevertheless, in all samples, *aw* values remained above the threshold commonly associated with fungal inhibition [48].

Also, acidification parameters (pH and TTA) showed limited changes throughout storage (Figure 3). As expected from the fermentation process, flatbreads containing fermented legume sourdoughs exhibited higher acidity than wheat and non-fermented legume formulations. At day 0, TTA values were markedly higher in fermented formulations, reaching 7.26 ± 0.18 mL in FLF_AM7_Grasspea compared with 2.90 ± 0.09 mL in CWF, while pH values were consistently lower. Conversely, CWF, CWF-P and non-fermented legume flatbreads showed higher initial pH values (5.81–5.94) and lower TTA values (2.80–3.20 mL) (Figure 3). During storage, all formulations exhibited a gradual pH decrease, becoming significant (*p* < 0.05) at day 60, together with a slight increase in TTA, which remained comparatively stable. This trend was also observed in the control formulations, including CWF, CWF-P and CLF samples, and may be partly attributed to CO_2_ dissolution from the MAP headspace into the bread moisture, resulting in carbonic acid formation, as previously described for CO_2_-enriched MAP systems [6].

#### 3.3.2. Microbial Stability and Visible Mold Monitoring

Microbiological fungal counts and visible mold incidence provide complementary information on fungal development and macroscopic spoilage, respectively. Visible mold spoilage in bakery products generally becomes evident only after substantial fungal proliferation, although the fungal population associated with visible spoilage can vary considerably (approximately 3–6 Log CFU g^−1^) depending on fungal species, substrate composition, water activity, and storage conditions [49,50,51].

The wheat flatbread containing calcium propionate (CWF-P), included as a positive preservative control, completely inhibited fungal development throughout the 60-day storage period, with fungal populations remaining below the detection limit (<2 Log CFU g^−1^) and no mold-positive packages detected (Figure 4 and Figure 5).

Conversely, control wheat flatbread (CWF) and all non-fermented legume formulations (CLF) showed rapid fungal development. In these samples, fungal populations increased after 15 days of storage, up to 3.2 Log CFU g^−1^ and progressively increasing to around 6 Log CFU g^−1^ by day 60. In parallel, the incidence of visible mold increased over time, with CLF_Pea showing the highest susceptibility, as visible mold was detected in 58% of packages after 35 days of storage.

Fermentation markedly improved fungal stability of legume-based flatbreads compared with the corresponding non-fermented formulations; however, the magnitude of the protective effect was strongly dependent on the legume substrate and starter combination and did not reach the complete fungal suppression observed with calcium propionate (CWF-P). FLF_AM7_Grasspea showed the highest antifungal performance, maintaining fungal populations below the detection limit (<2 Log CFU g^−1^) throughout the entire storage period (Figure 4), although visible mold was detected in 5% of packages at day 60 (Figure 5). Similarly, FLF_1A7_Pea maintained fungal counts below the detection limit (<2 log CFU g^−1^) up to day 45, followed by a limited increase to 2 log CFU g^−1^ at day 60, while visible mold remained restricted to 3% of packages (Figure 5). Fermented chickpea and lentil flatbreads also exhibited improved resistance to fungal spoilage compared with their corresponding non-fermented formulations, although the effect was less pronounced. FLF_1A7_Chickpea showed limited fungal development, reaching 2.97 ± 0.07 Log CFU g^−1^ at day 60, with visible mold detected in 12.4% of packages. Conversely, FLF_1A7_Lentil displayed a weaker protective effect, with fungal populations increasing to 3.90 ± 0.09 Log CFU g^−1^ and visible mold occurring in ca. 20% of packages at the end of storage.

Although visible mold growth represents the practical endpoint commonly used to define bakery product shelf life, fungal colonisation may precede macroscopic spoilage, and mycotoxin biosynthesis can potentially occur during early stages of fungal development before visible growth becomes evident [50,52,53]. Therefore, the prolonged suppression of fungal growth observed in FLF_AM7_Grasspea and FLF_1A7_Pea represents a relevant improvement in microbiological stability under MAP conditions.

Within the limits of the present dataset, the superior performance of these two formulations coincided with the highest release of free amino acids during sourdough fermentation (Table 3). This association is consistent with the possibility that nitrogen metabolism contributes to the production of antifungal metabolites. Phenyllactic acid (PLA) may represent one of the compounds potentially involved, as phenylalanine can serve as a precursor for PLA biosynthesis by lactic acid bacteria, including the species employed in this study [54]. PLA is a non-volatile organic acid with broad-spectrum antifungal activity and high thermal stability, properties that may favor its persistence during processing and contribute to fungal growth inhibition in fermented bakery products [41,54,55,56]. However, because individual compounds such as PLA, phenolic metabolites, bioactive peptides, and volatile organic compounds were not quantified in the present study, the mechanisms underlying the observed in situ protection remain to be elucidated.

#### 3.3.3. Texture Profile Monitoring

Bread hardness is widely recognized as a reliable instrumental indicator of staling and quality deterioration, reflecting structural changes within the crumb matrix mainly associated with starch retrogradation and moisture redistribution [57]. The incorporation of legume flours is known to markedly affect the textural properties of baked goods, as legume-based ingredients may impair gluten network development, increase competition for water, and modify starch–protein interactions, resulting in reduced crumb elasticity and increased firmness of baked products [58,59,60,61,62]. However, sourdough fermentation has been reported to partially mitigate these adverse effects through biochemical modifications, including acidification and proteolysis, which can influence dough structure and crumb properties [60]. As shown in Figure 6, at day 0 all legume-enriched flatbreads, including both fermented (FLF) and unfermented (CLF) formulations, exhibited significantly (*p* < 0.05) higher hardness values than wheat controls (CWF and CWF-P).

Among the tested formulations, CLF_Chickpea showed the highest initial hardness (22.96 ± 0.69 N), whereas fermented legume flatbreads generally exhibited lower (*p* < 0.05) hardness compared with their corresponding non-fermented counterparts. This effect was particularly evident for FLF_AM7_Grasspea, which displayed hardness values closer to those of wheat controls (CWF and CWF-P) (Figure 6). The lower hardness observed in fermented compared with non-fermented legume formulations may be related to the biochemical modifications occurring during sourdough fermentation. In particular, acidification and proteolysis may partially modify protein–starch and protein–water interactions within the dough matrix, potentially reducing the firmness of the resulting flatbreads [47]. During storage, all formulations showed a progressive increase in hardness, consistent with the typical evolution of bread staling driven by starch retrogradation and moisture redistribution, which progressively reduce crumb softness and elasticity [6,62,63]. Although modified atmosphere packaging can delay quality deterioration by limiting moisture loss and slowing physicochemical changes, it does not completely prevent firmness development during extended storage [6]. The magnitude of hardening differed among formulations: CLF_Chickpea remained the hardest product throughout storage, increasing from 22.96 ± 0.69 N at day 0 to 25.32 ± 0.41 N at day 15 and reaching 28.44 ± 0.82 N at day 60. Conversely, fermented formulations generally showed lower hardness values (*p* < 0.05) than their corresponding non-fermented counterparts at each sampling point. During the final storage stages (45 and 60 days), several FLF- and CLF-based flatbreads reached hardness values above 25 N, although this was not observed for all formulations. FLF_AM7_Grasspea generally showed the lowest hardness among the fermented legume formulations, remaining below 25 N up to day 60.

## 4. Conclusions

The present study provides pilot-scale evidence supporting the technological and industrial feasibility of using fermented legume ingredients as a clean-label strategy to improve the microbiological stability of legume-based flatbreads under modified atmosphere packaging. Under processing and storage conditions representative of industrial practice, selected fermentation systems were able to delay fungal spoilage while contributing to the maintenance of product texture, thus demonstrating that fermentation can be integrated into existing bakery production chains without compromising the technological requirements of the final product. These findings establish a proof of concept for fermented legume ingredients as multifunctional components with both preservative and technological functions. The variability among legume–starter combinations provides a basis for the rational optimization of fermentation systems through the characterization of matrix composition, starter metabolic traits and antifungal metabolites. Future studies based on metabolomic and targeted analytical approaches, combined with strain-level phenotypic characterization, could help identify the biochemical determinants underlying the most effective combinations. Overall, this integrated approach may enable the development of tailored fermentation systems designed to meet specific technological, shelf-life and market requirements for clean-label legume-based bakery products.

## Figures and Tables

**Figure 1:**
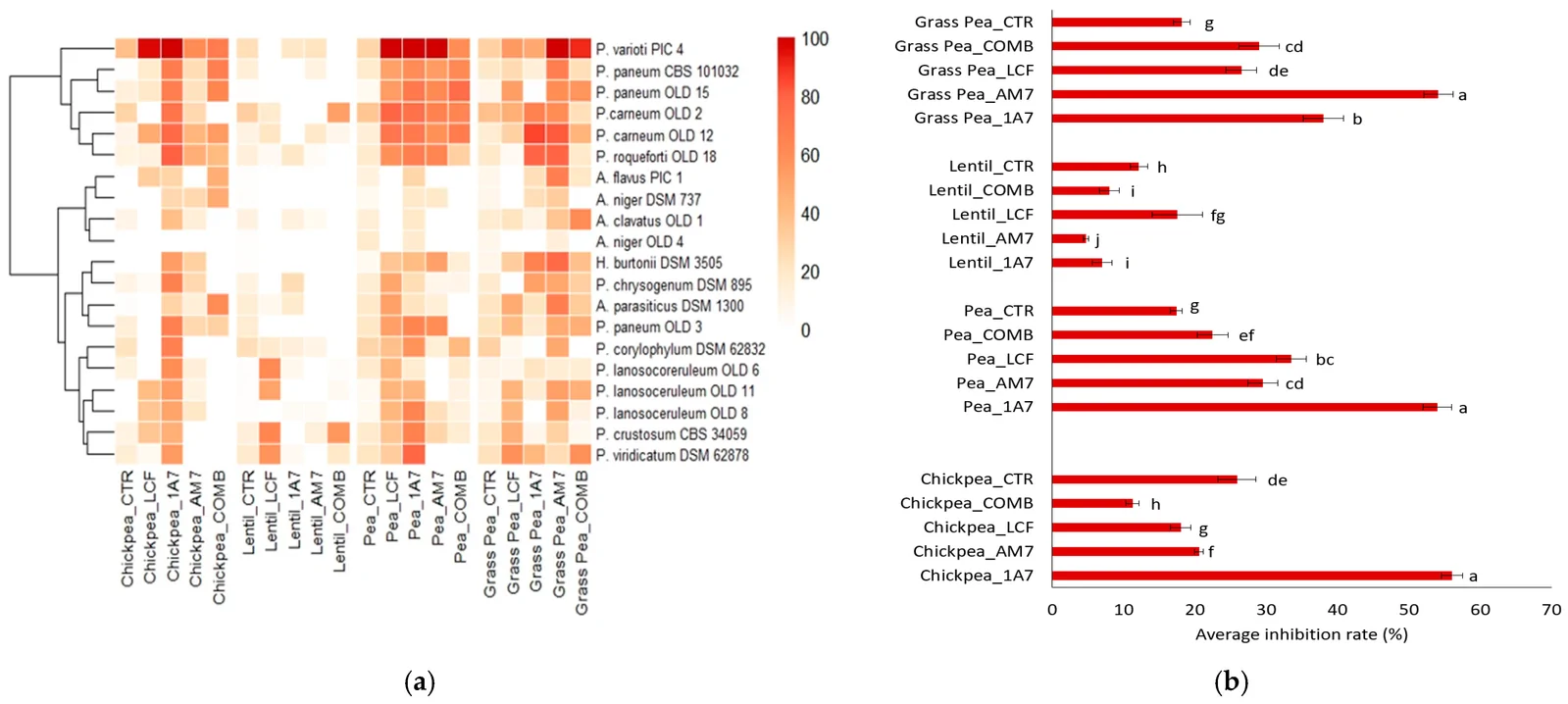
(**a**) Heatmap showing the percentage inhibition of fungal radial growth induced by water/salt-soluble (*w*LS) fractions obtained from legume-based sourdoughs. Rows represent fungal strains, and columns represent wLS fractions derived from chickpea, pea, lentil, and grass pea sourdoughs, including the corresponding unfermented controls (CTR). Colour intensity indicates inhibition levels (0–100%). The dendrogram represents hierarchical clustering of fungal strains according to similarity in their inhibition profiles. (**b**) Mean percentage radial growth inhibition exerted by each fermented legume-derived *w*LS fraction against the complete fungal strain collection. Values represent the average inhibition calculated across all tested fungal isolates. Different letters above bars indicate significant differences among treatments according to Tukey’s HSD test (*p* < 0.05).

**Figure 2:**
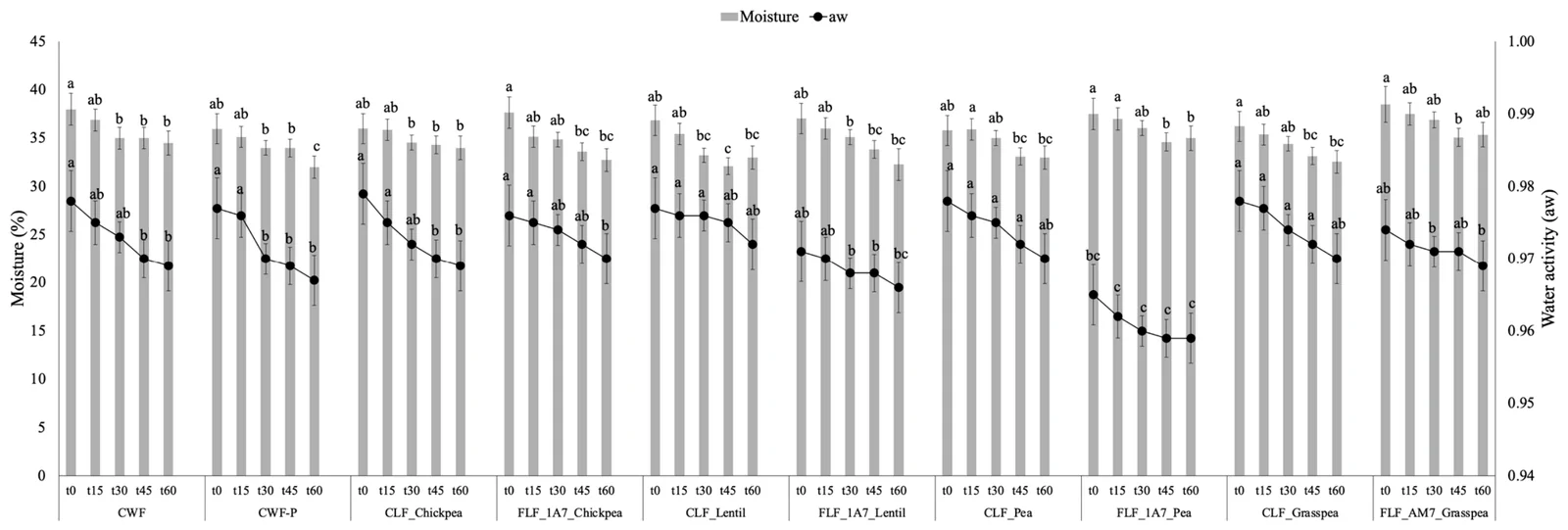
Changes in moisture content (%) (grey bars) and water activity (aw; black lines) of wheat- and legume-based focaccia-type flatbreads during storage under modified atmosphere packaging. Samples were analysed at 0, 15, 30, 45, and 60 days. CWF: control wheat focaccia; CWF-P: calcium propionate-containing wheat focaccia; CLF: control legume focaccia prepared with unfermented legume flour; FLF: fermented legume focaccia obtained from sourdoughs prepared with *Lp. plantarum* 1A7 (chickpea, pea, and lentil) and *Lev. brevis* AM7 (grass pea). Data are expressed as mean ± standard deviation (n = 2). Different letters indicate significant differences among all formulation–storage time combinations for the corresponding parameter (moisture content or aw) according to one-way ANOVA followed by Tukey’s HSD test (*p* < 0.05).

**Figure 3:**
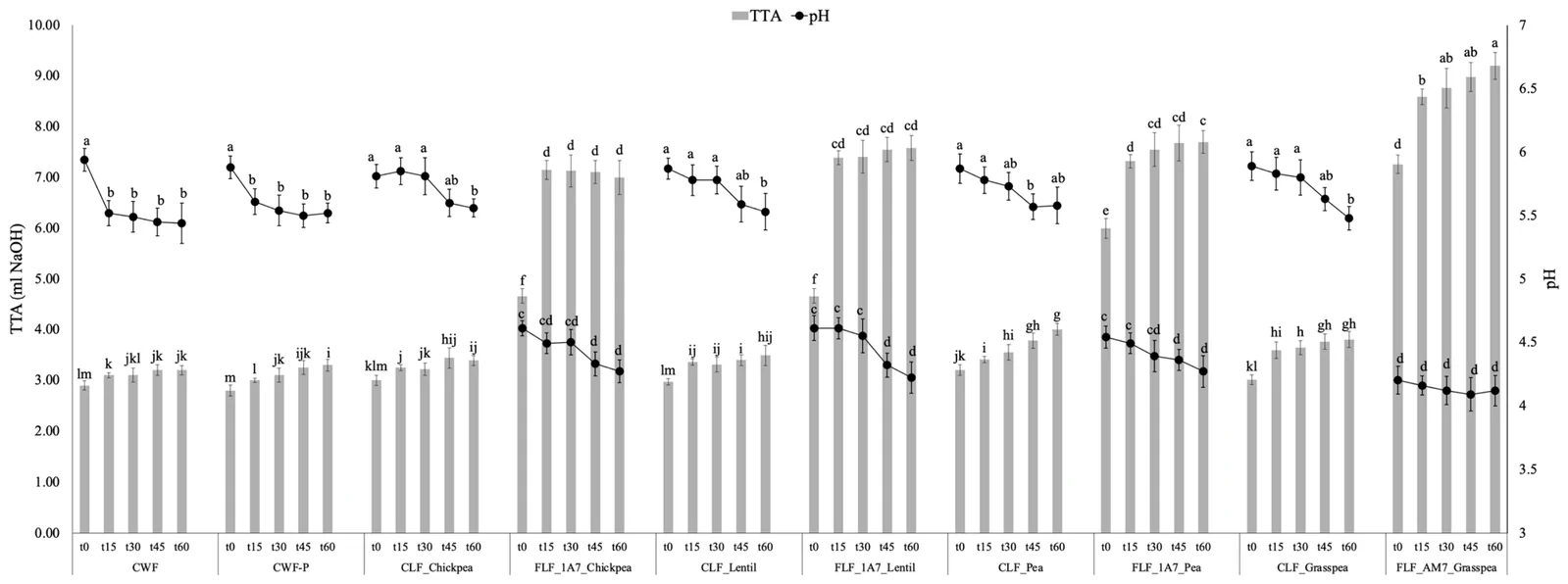
Changes in TTA (grey bars) and pH (black lines) of wheat- and legume-based focaccia-type flatbreads during storage under modified atmosphere packaging. Samples were analysed at 0, 15, 30, 45, and 60 days. CWF: control wheat focaccia; CWF-P: calcium propionate-containing wheat focaccia; CLF: control legume focaccia prepared with unfermented legume flour; FLF: fermented legume focaccia containing legume sourdoughs prepared with selected starters, including *Lp. plantarum* 1A7 (chickpea, pea, and lentil) and *Lev. brevis* AM7 (grass pea). Data are expressed as mean ± standard deviation (n = 2). Different letters indicate significant differences among all formulation–storage time combinations for the corresponding parameter (TTA or pH) according to one-way ANOVA followed by Tukey’s HSD test (*p* < 0.05).

**Figure 4:**
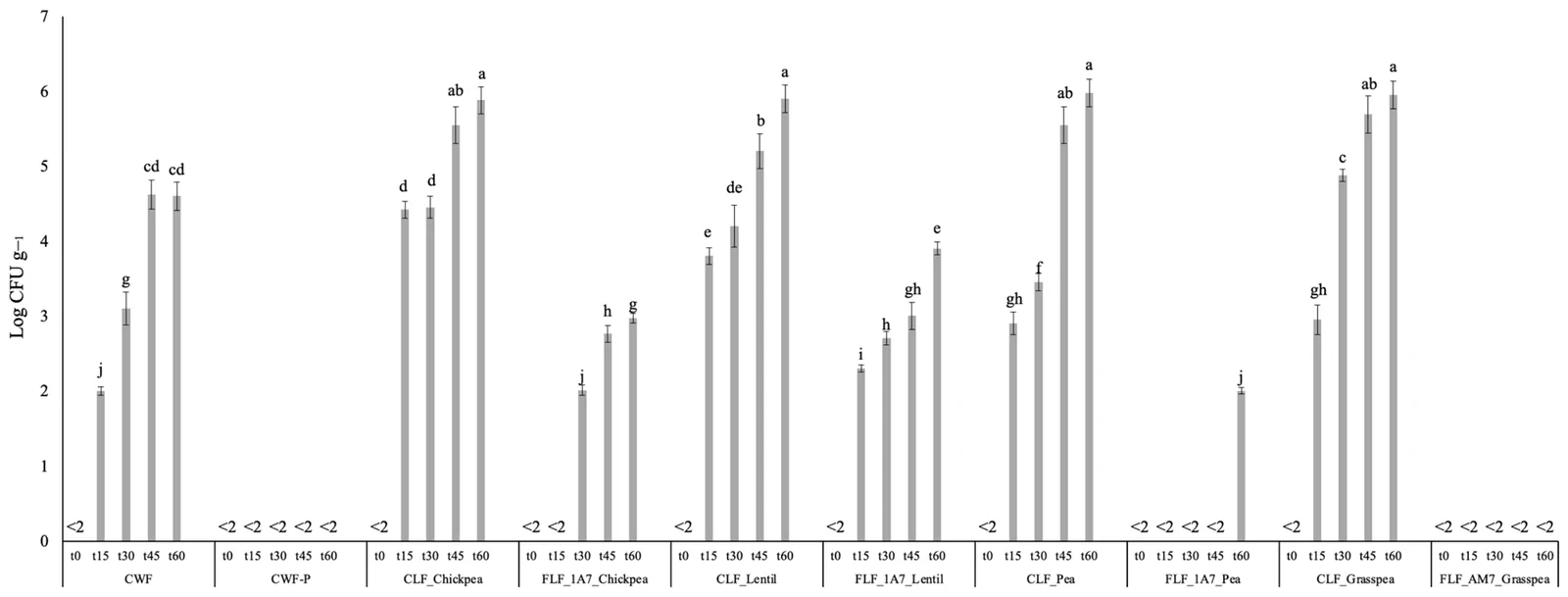
Changes in mold cell density (Log CFU g^−1^) of wheat- and legume-based focaccia-type flatbreads during storage under modified atmosphere packaging. Samples were analysed at 0, 15, 30, 45, and 60 days. CWF: control wheat focaccia; CWF-P: calcium propionate-containing wheat focaccia; CLF: control legume focaccia prepared with unfermented legume flour; FLF: fermented legume focaccia containing legume sourdoughs prepared with selected starters, including *Lp. plantarum* 1A7 (chickpea, pea, and lentil) and *Lev. brevis* AM7 (grass pea). The detection limit was 2 Log CFU g^−1^; values below this limit are reported as <2 Log CFU g^−1^. Data are expressed as mean ± standard deviation (n = 2). Different letters indicate significant differences among formulation–storage time combinations according to one-way ANOVA followed by Tukey’s HSD test (*p* < 0.05).

**Figure 5:**
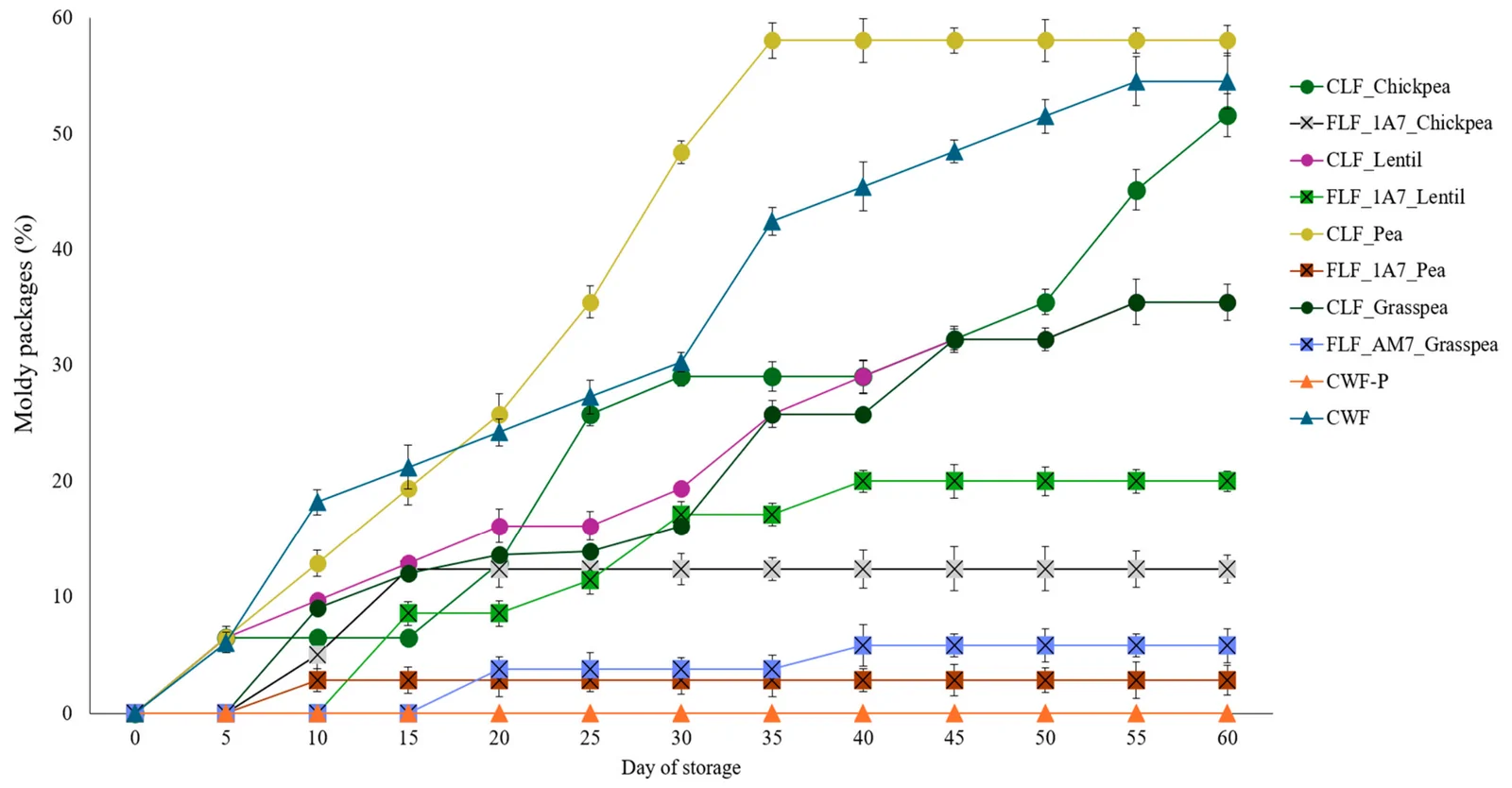
Visible mold incidence (%) in wheat- and legume-based focaccia-type flatbreads during 60 days of storage under modified atmosphere packaging. CWF: control wheat focaccia; CWF-P: calcium propionate-containing wheat focaccia; CLF: control legume focaccia prepared with unfermented legume flour; FLF: fermented legume focaccia containing legume sourdoughs prepared with selected starters, including *Lp. plantarum* 1A7 (chickpea, pea, and lentil) and *Lev. brevis* AM7 (grass pea). Values represent the mean ± SD of two independent production trials (n = 2), with 25 unopened packages continuously monitored per trial (50 packages per formulation in total).

**Figure 6:**
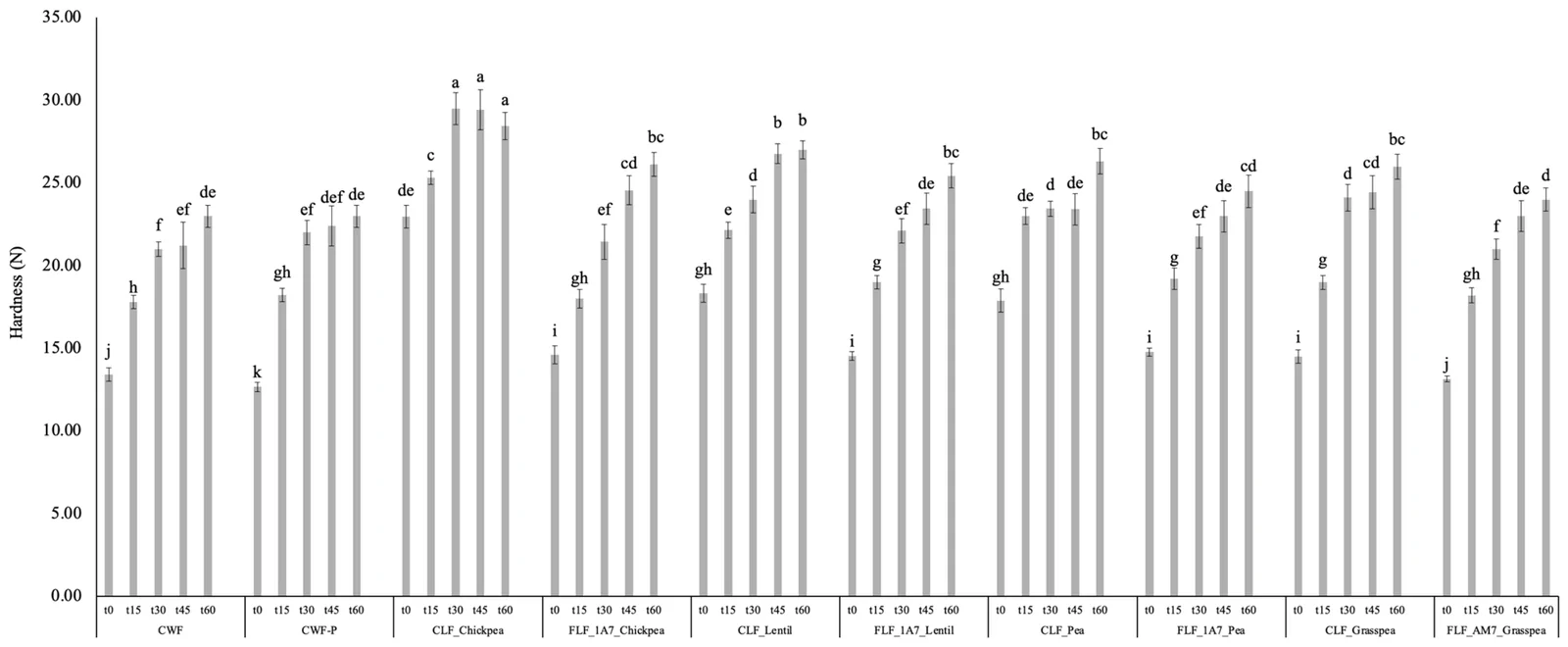
Hardness (N) changes in wheat- and legume-based focaccia-type flatbreads for 60 days storage under modified atmosphere packaging. CWF: control wheat focaccia; CWF-P: calcium propionate-containing wheat focaccia; CLF: control legume focaccia prepared with unfermented legume flour; FLF: fermented legume focaccia containing legume sourdoughs prepared with selected starters, including *Lp. plantarum* 1A7 (chickpea, pea, and lentil) and *Lev. brevis* AM7 (grass pea). Data are expressed as mean ± standard deviation (n = 2). Different letters indicate significant differences among formulation–storage time combinations according to one-way ANOVA followed by Tukey’s HSD test (*p* < 0.05).

**Table 1:** Fungal species, strain codes, and culture collections of the microorganisms used in this study. Strains include isolates from internal collections (Department of Soil, Plant and Food Sciences; Vallefiorita S.r.l.) and reference culture collections (CBS-KNAW Culture Collection and DSMZ—Leibniz Institute DSMZ–German Collection of Microorganisms and Cell Cultures GmbH).

Fungal Species	Strain Code	Culture Collection
*Aspergillus clavatus*	OLD 1	Department of Soil, Plant and Food Sciences
*Aspergillus niger*	OLD 4	
*Penicillium carneum*	OLD 2	
*Penicillium carneum*	OLD 12	
*Penicillium lanosocoeruleum*	OLD 6	
*Penicillium lanosocoeruleum*	OLD 8	
*Penicillium lanosocoeruleum*	OLD 11	
*Penicillium paneum*	OLD 3	
*Penicillium paneum*	OLD 15	
*Penicillium roqueforti*	OLD 18	
*Aspergillus flavus*	PIC 1	Vallefiorita S.r.l.
*Paecilomyces variotii*	PIC 4	
*Penicillium crustosum*	CBS 340.59	CBS-KNAW Culture Collection
*Penicillium paneum*	CBS 101032	
*Aspergillus niger*	DSM 737	Leibniz Institute DSMZ—German Collection of Microorganisms and Cell Cultures GmbH (Braunschweig, Germany)
*Aspergillus parasiticus*	DSM 1300	
*Penicillium chrysogenum*	DSM 895	
*Penicillium corylophilum*	DSM 62832	
*Penicillium viridicatum*	DSM 62878	
*Hyphopichia burtonii*	DSM 3505	

**Table 2:** Formulations of focaccia-type flatbreads expressed as % *w*/*w* on dough basis (d.b.) and flour basis (f.b.). CWF, control wheat focaccia; CWF-P, wheat focaccia supplemented with calcium propionate (0.3%, *w*/*w*, flour basis); FLF, focaccia containing selected legume sourdoughs (chickpea, lentil, pea, or grass pea) at 33% (*w*/*w*) of total dough weight; CLF, focaccia containing the corresponding unfermented legume flours at the same legume-derived dry matter contribution as FLF formulations. All doughs were standardized to a final dough yield (DY) of 153.

	CWF		CWF-P		FLF		CLF	
	% d.b.	% f.b.	% d.b.	% f.b.	% d.b. ^a^	% f.b. ^a^	% d.b.	% f.b.
Wheat flour (type 0)	32.5	50	32.5	50	27.5	42.3	27.5	42.3
Durum wheat semolina	32.5	50	32.5	50	27.5	42.3	27.5	42.3
Water	35.0	53	35.0	53	12	18	35	53
Legume flour	–	**–**	–	**–**	–	–	10	15.3
Legume sourdough	–	**–**	–	**–**	33	–	–	–
Legume sourdough composition								
Legume flour	–	**–**	–	**–**	10	15.3	–	–
Water	–	**–**	–	**–**	23	35	–	–
Baker’s yeast *	1.8	2.8	1.8	2.8	1.8	2.8	1.8	2.8
Barley malt *	0.6	1.0	0.6	1.0	0.6	1.0	0.6	1.0
Salt *	1.6	2.5	1.6	2.5	1.6	2.5	1.6	2.5
Extra virgin olive oil *	2.4	3.7	2.4	3.7	2.4	3.7	2.4	3.7
Calcium propionate	–	**–**	0.2	0.3	–	**–**	–	**–**
Flour sum ^a^	65	100	65	100	65	100	65	100
Water sum ^b^	35	53	35	53	35	53	35	53

^a^ Flour sum was calculated as the sum of wheat flour, durum wheat semolina and legume flour supplied by legume sourdough (or the corresponding non-fermented legume flour in control formulations). The total flour content was normalized to 100% flour basis (f.b.). ^b^ Water sum was calculated as the sum of water added during dough mixing and the water supplied by the fermented legume sourdough, maintaining identical total flour and water contents. * Additional ingredients were not included into the dough yield calculation.

**Table 3:** Microbiological, chemical, and biochemical parameters of legume-based doughs (DY 330: chickpea, lentil, pea, and grass pea) inoculated with starter cultures at 7 Log CFU g^−1^ for lactic acid bacteria and 6 Log CFU g^−1^ for yeasts, and analyzed before (0 h) and after (24 h) fermentation. The LAB starters included 1A7 (*Lactiplantibacillus plantarum*) and AM7 (*Levilactobacillus brevis*), while the yeast starter was *Wickerhamomyces anomalus* LCF1695 (LCF). A mixed starter culture (COMB) consisted of all three strains.

Flour	Strain	MRS (Log CFU g^−1^)		MEA (Log CFU g^−1^)		pH		TTA (mL NaOH)		Lactic Acid (mmol/kg)		Acetic Acid (mmol/kg)		Peptides (mg/g)		TFAA (g/kg)	
		0 h	24 h	0 h	24 h	0 h	24 h	0 h	24 h	0 h	24 h	0 h	24 h	0 h	24 h	0 h	24 h
Chickpea	1A7	7.17 ± 0.11 ^a,B^	9.73 ± 0.21 ^a,A^	n.d	n.d	6.60 ± 0.13 ^a,A^	4.06 ± 0.12 ^de,B^	2.43 ± 0.10 ^c,B^	16.40 ± 0.33 ^cd,A^	2.58 ± 0.05 ^a,B^	108.39 ± 2.71 ^ab,A^	2.03 ± 0.06 ^a,B^	19.81 ± 0.44 ^a,A^	25.78 ± 1.03 ^cd,A^	22.02 ± 0.66 ^f,B^	1.20 ± 0.03 ^e,A^	1.10 ± 0.02 ^g,B^
	AM7	7.14 ± 0.18 ^a,B^	9.67 ± 0.15 ^a,A^	n.d	n.d	6.58 ± 0.19 ^a,A^	3.53 ± 0.11 ^f,B^	2.47 ± 0.08 ^c,B^	17.63 ± 0.49 ^bc,A^	2.63 ± 0.08 ^a,B^	107.44 ± 1.83 ^b,A^	2.08 ± 0.04 ^a,B^	14.52 ± 0.49 ^b,A^	27.17 ± 0.66 ^c,A^	12.22 ± 0.39 ^i,B^	1.27 ± 0.03 ^d,B^	3.00 ± 0.12 ^c,A^
	LCF	4.32 ± 0.13 ^d,B^	6.36 ± 0.29 ^cd,A^	5.90 ± 0.31 ^a,B^	8.22 ± 0.17 ^b,A^	6.62 ± 0.22 ^a,A^	4.70 ± 0.13 ^b,B^	2.51 ± 0.06 ^c,B^	9.20 ± 0.27 ^f,A^	2.51 ± 0.06 ^a,B^	89.99 ± 3.15 ^d,A^	2.04 ± 0.07 ^a,B^	6.58 ± 0.29 ^h,A^	26.48 ± 0.64 ^c,A^	24.59 ± 0.89 ^e,B^	1.12 ± 0.02 ^f,A^	1.04 ± 0.04 ^g,B^
	COMB	7.10 ± 0.39 ^a,B^	9.20 ± 0.46 ^a,A^	6.20 ± 0.37 ^a,B^	8.23 ± 0.46 ^ab,A^	6.55 ± 0.18 ^a,A^	4.10 ± 0.12 ^d,B^	2.42 ± 0.07 ^c,B^	13.40 ± 0.50 ^e,A^	2.70 ± 0.11 ^a,B^	108.12 ± 2.81 ^ab,A^	2.10 ± 0.05 ^a,B^	12.99 ± 0.29 ^c,A^	24.81 ± 0.39 ^d,A^	14.12 ± 0.35 ^h,B^	0.85 ± 0.02 ^g,A^	0.51 ± 0.01 ^h,B^
Lentil	1A7	7.32 ± 0.11 ^a,B^	9.73 ± 0.14 ^a,A^	n.d	n.d	6.48 ± 0.28 ^a,A^	4.16 ± 0.08 ^cd,B^	3.63 ± 0.16 ^ab,B^	18.60 ± 0.32 ^a,A^	0.57 ± 0.02 ^c,B^	105.94 ± 4.66 ^b,A^	1.35 ± 0.06 ^b,B^	12.99 ± 0.25 ^c,A^	40.59 ± 1.01 ^a,A^	35.50 ± 1.42 ^b,B^	1.68 ± 0.06 ^c,B^	3.12 ± 0.14 ^bc,A^
	AM7	7.28 ± 0.16 ^a,B^	9.91 ± 0.27 ^a,A^	n.d	n.d	6.55 ± 0.20 ^a,A^	3.53 ± 0.15 ^f,B^	3.65 ± 0.08 ^ab,B^	16.17 ± 0.71 ^d,A^	0.60 ± 0.02 ^c,B^	112.96 ± 2.15 ^a,A^	1.38 ± 0.04 ^b,B^	9.54 ± 0.42 ^f,A^	42.32 ± 1.10 ^a,A^	24.14 ± 1.01 ^e,B^	1.64 ± 0.08 ^c,B^	3.28 ± 0.09 ^b,A^
	LCF	5.12 ± 0.11 ^b,B^	7.12 ± 0.14 ^b,A^	5.93 ± 0.33 ^a,B^	8.72 ± 0.17 ^a,A^	6.49 ± 0.38 ^a,A^	4.65 ± 0.10 ^b,B^	3.60 ± 0.13 ^ab,B^	8.23 ± 0.42 ^g,A^	0.61 ± 0.02 ^c,B^	95.92 ± 2.97 ^cd,A^	1.32 ± 0.05 ^b,B^	5.45 ± 0.12 ^i,A^	40.65 ± 1.38 ^a,A^	37.58 ± 0.83 ^b,B^	1.56 ± 0.04 ^c,B^	1.82 ± 0.09 ^f,A^
	COMB	7.17 ± 0.37 ^a,B^	9.57 ± 0.48 ^a,A^	6.35 ± 0.36 ^a,B^	8.66 ± 0.46 ^ab,A^	6.50 ± 0.12 ^a,A^	4.20 ± 0.15 ^cd,B^	3.70 ± 0.04 ^a,B^	13.60 ± 0.35 ^e,A^	0.58 ± 0.02 ^c,B^	107.64 ± 3.55 ^ab,A^	1.34 ± 0.05 ^b,,B^	7.94 ± 0.36 ^g,A^	41.19 ± 1.00 ^a,A^	28.25 ± 1.16 ^d,B^	1.60 ± 0.03 ^c,B^	3.34 ± 0.15 ^b,A^
Pea	1A7	7.13 ± 0.22 ^a,B^	9.64 ± 0.31 ^a,A^	n.d	n.d	6.49 ± 0.16 ^a,A^	4.09 ± 0.08 ^d,B^	3.63 ± 0.16 ^ab,B^	18.80 ± 0.73 ^ab,A^	n.d	107.37 ± 2.36 ^b,A^	0.68 ± 0.02 ^c,B^	15.24 ± 0.62 ^b,A^	37.36 ± 1.68 ^b,A^	32.62 ± 0.85 ^c,B^	2.58 ± 0.08 ^a,B^	5.95 ± 0.29 ^a,A^
	AM7	7.28 ± 0.15 ^a,B^	9.55 ± 0.17 ^a,A^	n.d	n.d	6.51 ± 0.20 ^a,A^	3.49 ± 0.17 ^f,B^	3.66 ± 0.12 ^ab,B^	18.50 ± 0.31 ^a,A^	n.d	109.14 ± 4.80 ^ab,A^	0.64 ± 0.02 ^c,B^	12.11 ± 0.27 ^d,A^	36.56 ± 1.21 ^b,A^	20.24 ± 0.87 ^g,B^	2.50 ± 0.12 ^a,B^	2.99 ± 0.10 ^c,A^
	LCF	4.78 ± 0.13 ^c,B^	6.78 ± 0.16 ^c,A^	6.14 ± 0.10 ^a,B^	8.43 ± 0.24 ^ab,A^	6.50 ± 0.28 ^a,A^	5.07 ± 0.19 ^a,B^	3.65 ± 0.09 ^ab,B^	8.40 ± 0.41 ^g,A^	n.d	96.87 ± 3.00 ^c,A^	0.70 ± 0.02 ^c,B^	3.53 ± 0.11 ^j,A^	35.88 ± 0.93 ^b,B^	43.82 ± 0.92 ^a,A^	2.63 ± 0.13 ^a,A^	2.40 ± 0.11 ^e,B^
	COMB	7.30 ± 0.39 ^a,B^	9.34 ± 0.47 ^a,A^	6.09 ± 0.21 ^a,B^	8.44 ± 0.46 ^ab,A^	6.47 ± 0.14 ^a,A^	4.30 ± 0.06 ^c,B^	3.60 ± 0.07 ^ab,B^	16.80 ± 0.59 ^cd,A^	n.d	108.19 ± 3.14 ^ab,A^	0.62 ± 0.02 ^c,B^	9.95 ± 0.22 ^f,A^	35.98 ± 0.99 ^b,A^	27.83 ± 0.89 ^d,B^	2.65 ± 0.06 ^a,B^	2.78 ± 0.06 ^d,A^
Grass pea	1A7	7.13 ± 0.16 ^a,B^	9.45 ± 0.29 ^a,A^	n.d	n.d	6.40 ± 0.15 ^a,A^	4.15 ± 0.11 ^e,B^	3.43 ± 0.16 ^b,B^	18.67 ± 0.78 ^ab,A^	2.01 ± 0.08 ^b,B^	107.50 ± 2.37 ^b,A^	n.d	14.76 ± 0.32 ^b,A^	36.56 ± 0.69 ^b,A^	35.87 ± 1.58 ^b,A^	1.88 ± 0.05 ^b,B^	3.37 ± 0.15 ^b,A^
	AM7	7.00 ± 0.25 ^a,B^	9.61 ± 0.21 ^a,A^	n.d	n.d	6.42 ± 0.22 ^a,A^	3.46 ± 0.12 ^f,B^	3.41 ± 0.12 ^b,B^	18.70 ± 0.34 ^a,A^	2.15 ± 0.06 ^b,B^	108.05 ± 3.35 ^ab,A^	n.d	11,15 ± 0.46 ^e,A^	35.70 ± 0.79 ^b,A^	29.89 ± 1.26 ^d,B^	1.93 ± 0.07 ^b,B^	6.47 ± 0.23 ^a,A^
	LCF	4.33 ± 0.17 ^d,B^	6.25 ± 0.19 ^d,A^	5.93 ± 0.23 ^a,B^	8.76 ± 0.33 ^a,A^	6.44 ± 0.26 ^a,A^	4.47 ± 0.10 ^b,B^	3.40 ± 0.05 ^b,B^	9.11 ± 0.34 ^fg,A^	2.20 ± 0.10 ^b,B^	92.51 ± 3.89 ^cd,A^	n.d	2.89 ± 0.09 ^k,A^	36.02 ± 1.30 ^b,A^	24.65 ± 0.44 ^e,B^	1.84 ± 0.05 ^b,A^	1.79 ± 0.06 ^f,B^
	COMB	7.18 ± 0.39 ^a,B^	9.41 ± 0.47 ^a,A^	6.01 ± 0.37 ^a,B^	8.36 ± 0.47 ^ab,A^	6.39 ± 0.23 ^a,A^	4.10 ± 0.11 ^d,B^	3.60 ± 0.07 ^ab,B^	15.80 ± 0.49 ^d,A^	2.05 ± 0.07 ^b,B^	108.46 ± 3.58 ^ab,A^	n.d	11.15 ± 0.49 ^e,A^	36.03 ± 1.27 ^b,A^	27.41 ± 0.90 ^d,B^	1.90 ± 0.03 ^b,B^	2.30 ± 0.06 ^e,A^

MRS: de Man, Rogosa and Sharpe agar; MEA: malt extract agar; TTA: total titratable acidity; TFAA: total free amino acids. n.d.: not detected. Data are expressed as mean ± standard deviation (n = 3). Different lowercase letters (a–k) within the same column indicate significant differences among samples at the same time point, whereas different uppercase letters (A–B) within the same row indicate significant differences between 0 h and 24 h for the same sample (ANOVA followed by Tukey’s HSD test, *p* < 0.05).

## Data Availability

The original contributions presented in this study are included in the article and Supplementary Materials. Further inquiries can be directed to the corresponding author.

## Supplementary Materials

The following supporting information can be downloaded at: https://www.mdpi.com/article/10.3390/foods15193368/s1, Table S1. Proximate composition and energy values of selected legumes reported on a dry matter basis, excluding moisture content, according to manufacturer specifications.
