# Supplementary material for: Fermented Legumes as a Clean-Label Strategy for Shelf-Life Extension of Flatbread Under Modified Atmosphere Packaging: A Pilot-Scale Validation
Source: Foods. 2026 Sep 22;15(19):3368. doi: 10.3390/foods15193368 (PMC13664338; doi:10.3390/foods15193368)
Supplement: Supplementary file 1 [file foods-15-03368-s001.zip › foods-4533780-supplementary.pdf]

Table S1. Proximate composition and energy values of selected legumes reported on a dry matter basis, excluding moisture content, according to manufacturer specifications.

Legume	Energy (kcal/100 g)	Protein (g/100 g)	Carbohydrates (g/100 g)	Lipids (g/100 g)	Dietary fiber (g/100 g)	Energy (kcal/100 g)
Cicer arietinum (Chickpea)	316	21.8	47.2	6.0	13.8	316
Lens culinaris (Lentil)	291	23.0	51.1	1.0	13.8	291
Pisum sativum (Pea)	303	21.7	48.5	1.5	15.7	303
Lathyrus sativus (Grass pea)	343	28.0	57.0	0.6	7.0	343
